# Factors Associated with Delayed Diagnosis of Iron Deficiency Anemia in Egyptian Patients: A Retrospective Cohort Study

**DOI:** 10.3390/healthcare14142112

**Published:** 2026-07-14

**Authors:** May AlMoshary, Ebtisam Bakhsh, Hadeer Ahmed Ali Esmaeil, Nahid Abdulhamid Qushmaq, Ahmad Ali Alharbi, Ekremah A. Alzarea, Ezeldine K. Abdalhabib, Mubarak Salem AlGhamdi

**Affiliations:** 1 Department of Basic Science, College of Medicine, Princess Nourah Bint Abdulrahman University, P.O. Box 84428, Riyadh 11671, Saudi Arabia; hemmai2020@yahoo.com; 2 Department of Internal Medicine, Princess Nourah bint Abdulrahman University, Riyadh 11671, Saudi Arabia; 3 Faculty of Medicine, Minia University, Minia 2431436, Egypt; hadeer3495@gmail.com; 4 King Abdullah Medical Complex (MCSH), Jeddah Second Cluster, Jeddah 23816, Saudi Arabia; nqushmaq@moh.gov.sa; 5 Department of Basic Medical Sciences, College of Medicine, Majmaah University, Majmaah 11952, Saudi Arabia; aalharbi@mu.edu.sa; 6 Hematopathology, Department of Pathology, College of Medicine, Jouf University, Sakaka 72388, Saudi Arabia; eaalzare@ju.edu.sa; 7 Department of Clinical Laboratory Sciences, College of Applied Medical Sciences–AlQurayyat, Jouf University, Sakaka 72388, Saudi Arabia; ezeldine@ju.edu.sa; 8 Department of Internal Medicine & Critical Care, King Abdullah Bin Abdulaziz University Hospital, Princess Nourah Bint Abdulrahman University, Riyadh 11671, Saudi Arabia; msalghamdi@kaauh.edu.sa

**Keywords:** iron deficiency anemia, diagnostic delay, Egypt, retrospective cohort, Cox regression, healthcare system, MENA

## Abstract

**Highlights:**

**What are the main findings?**

- Nearly half (44.3%) of Egyptian IDA patients experienced a diagnostic delay exceeding 90 days, with a median primary delay of 76 days.
- Urban residence was independently associated with slower diagnostic confirmation in multivariable Cox regression.

**What are the implications of the main findings?**

- Healthcare fragmentation in Egyptian urban settings and laboratory inefficiencies are key modifiable targets to reduce IDA diagnostic delay.
- Structured primary care pathways and public health education on early IDA symptom recognition are urgently needed.

**Abstract:**

**Background/Objectives:** Iron deficiency anemia (IDA) remains the most prevalent nutritional deficiency worldwide and a leading cause of years lived with disability, particularly in low-income countries. In Egypt, the burden is substantial and compounded by regional socioeconomic, nutritional, and parasitic factors. Despite this, the predictors of diagnostic delay within the Egyptian healthcare system remain poorly characterized. This study aimed to quantify the extent of diagnostic delay among Egyptian patients with IDA and to identify the sociodemographic and clinical risk factors independently associated with prolonged time to diagnostic confirmation. **Methods:** A single-center retrospective cohort study was conducted at Al-Minia University Hospital, Egypt, between March and April 2026, enrolling 350 adults with confirmed IDA diagnosed between 2015 and 2025. “Diagnostic delay” was defined as the interval in days from the index date (the first documented abnormal complete blood count) to laboratory confirmation of iron deficiency by serum ferritin and/or transferrin saturation. Secondary outcomes included patient delay, system/testing delay, and etiology work-up delay. Time-to-event analysis was performed using Kaplan–Meier curves and Cox proportional hazards regression. All analyses were conducted in R version 4.4.0. **Results:** The cohort comprised 213 females (60.9%) and 137 males (39.1%), with a median age of 39 and 36 years, respectively. The median primary diagnostic delay was 76.0 days (IQR: 38.0, 182.8), with patient delay accounting for a median of 26.5 days and system/testing delay for 21.0 days. Overall, 155 patients (44.3%) experienced a diagnostic delay of more than 90 days. In the multivariable Cox model, urban residence (aHR = 0.69, 95% CI [0.53, 0.99], *p* = 0.045) was independently associated with a slower rate of diagnostic confirmation. **Conclusions:** Diagnostic delay in IDA is substantial in Egypt, driven by both patient-level symptom normalization and counterintuitive systemic bottlenecks. Urban residence was independently associated with prolonged time to diagnostic confirmation; ordering of iron studies at the first visit was associated with delay in bivariate analysis only and was not an independent predictor after multivariable adjustment. Targeted public health education, restructured primary care pathways, and improved laboratory turnaround times are essential to reduce this burden.

## 1. Introduction

Iron deficiency anemia (IDA) is considered the most common nutritional deficiency worldwide, affecting over 1.2 billion people and accounting for about half of all anemia cases globally [1,2]. IDA is still a leading cause of years lived with disability (YLDs), especially in low-income countries, despite improvements in diagnostic hematology and the availability of affordable therapies [3]. Its systemic effects are extensive, ranging from poor pregnancy outcomes and higher cardiovascular burden to impaired cognitive development and decreased physical work capability [4,5].

IDA is a serious public health issue in Egypt. National surveys and epidemiological data show consistently high prevalence across a range of demographic categories, especially among women of reproductive age, children, and adolescents [6,7]. Despite two decades of expanded iron supplementation and food fortification programs led by Egypt’s Ministry of Health, the population-level burden remains high, compounded by socioeconomic disparities, shifts toward low-bioavailability diets, and endemic intestinal parasitic infections that impair iron absorption [8,9].

Diagnostic delay, broadly defined as the interval between the onset of a biochemically or clinically detectable abnormality and the initiation of confirmed, appropriate treatment, has been operationalized in the hematology and primary care literature using a three-interval model comprising the patient interval (symptom onset to first healthcare contact), the system interval (first contact to confirmed diagnosis), and the total diagnostic interval [10,11]. Studies from high-income settings have documented that primary care physicians frequently defer iron studies in patients presenting with non-specific fatigue, particularly in younger adults and women of reproductive age, contributing to protracted system intervals [12,13]. However, these findings derive predominantly from well-resourced healthcare systems with unified electronic records and standardized referral pathways, and their applicability to fragmented, resource-constrained settings such as Egypt remains uncertain.

Even though IDA is quite common throughout the Middle East and North Africa (MENA) region, there is a lack of information on the factors that influence diagnostic delay in Egypt’s healthcare system. Prior Egyptian research has focused predominantly on IDA prevalence and nutritional interventions [14,15]. To improve screening procedures and patient outcomes, it is crucial to identify these factors, which include socioeconomic status, geographic area, symptom presentation, and the level of healthcare provision [11].

This retrospective cohort study was conducted to quantify diagnostic delay among Egyptian patients with IDA and to identify its sociodemographic and clinical risk factors. The findings aim to support evidence-based recommendations for health policy and clinical guidelines within the Egyptian context.

## 2. Materials and Methods

### 2.1. Study Design and Setting

This single-center, retrospective cohort study was conducted at Al-Minia University Hospital, Egypt, and is reported in accordance with the STROBE (Strengthening the Reporting of Observational Studies in Epidemiology) guidelines for cohort studies [16]. Between March and April 2026, investigators retrospectively identified eligible patients previously diagnosed with IDA between 2015 and 2025. Face-to-face interviews were conducted exclusively to collect patient-reported data, including symptom onset date, initial point of healthcare contact, and dietary history, while all laboratory-based timelines, including the Index Date and the date of confirmatory iron studies, were extracted from institutional medical records. To minimize recall bias for objective outcomes, laboratory records were prioritized over patient recall wherever both sources were available, and discrepancies were resolved in favor of the documented record. The final enrolled cohort of 350 represents those who met full eligibility criteria and completed both the interview and record review.

### 2.2. Ethical Approval

This retrospective cohort study was conducted in accordance with the ethical principles of the Declaration of Helsinki (1975, revised in 2013). Ethical approval was obtained from the Institutional Review Board (IRB), Faculty of Medicine, Minia University, Egypt (Approval No. 921/03/2026; Approval Date: 9 March 2026). Patient confidentiality and data privacy were strictly maintained throughout the study, and all collected data were anonymized before analysis.

### 2.3. Study Population

#### 2.3.1. Eligibility Criteria

Inclusion criteria consisted of adults (≥18 years) at the time of IDA diagnosis; confirmed IDA based on WHO diagnostic criteria (hemoglobin below 12.0 g/dL in women or below 13.0 g/dL in men, combined with serum ferritin below 30 ng/mL or transferrin saturation below 16%); a clearly documented Index Date (date of the first abnormal complete blood count [CBC] showing low hemoglobin with low mean corpuscular volume [MCV, <80 fL] and/or low mean corpuscular hemoglobin [MCH, <27 pg]); a subsequent date of confirmatory iron studies in the medical record; a diagnosis confirmed between 2015 and 2025; and availability of sufficient medical records documenting the timeline of symptoms, laboratory investigations, and healthcare contacts.

We excluded patients with anemia of chronic disease without concurrent iron deficiency or mixed cases combining iron deficiency anemia and anemia of chronic disease that could not be reliably classified in a retrospective design, as such cases would compromise diagnostic clarity; patients with confirmed thalassemia trait or other hemoglobinopathies, given that these conditions independently produce low MCV and MCH values and would confound the laboratory-based Index Date definition; patients with anemia attributable to other etiologies, including vitamin B12 or folate deficiency; patients with documented active bleeding at the time of diagnosis, such as acute trauma or surgical hemorrhage; women who were pregnant at the time of initial IDA diagnosis, as pregnancy induces physiological changes in blood volume that alter standard hemoglobin thresholds and iron metabolism; and patients with incomplete medical records precluding reliable ascertainment of the Index Date or the diagnostic timeline.

#### 2.3.2. Recruitment, Screening, and Selection

In response to the reviewers’ request for greater transparency about the recruitment process, this subsection has been added to describe the flow of patients from initial identification to final enrollment and to address the potential for selection bias arising from non-participation. Eligible patients were identified by screening the hospital’s hematology and internal medicine laboratory and clinic databases for a recorded diagnosis of IDA between 2015 and 2025. Of 478 records initially identified as potentially eligible, 71 did not meet the laboratory or clinical eligibility criteria described in Section 2.3.1 (most commonly a mixed anemia picture, a confirmed hemoglobinopathy, or an undocumented or incomplete index date/diagnostic timeline) and were excluded at the chart-review stage. Among the remaining 407 eligible patients, 17 could not be reached for the required face-to-face interview, and 40 declined to participate when contacted. The final analytic cohort comprised the 350 patients who met all eligibility criteria and completed both the interview and the record review.

The 71 patients excluded at chart review did not meet the pre-specified eligibility criteria (Section 2.3.1) and are not considered a source of selection bias in the usual sense. However, sociodemographic and clinical data for the 57 eligible patients who could not be reached (17) or who declined participation (40) were not systematically recorded in a form available for analysis; therefore, a formal comparison between included and these non-participating eligible patients on age, sex, or other characteristics was not feasible, and residual selection bias arising from non-participation cannot be excluded.

### 2.4. Data Collection and Study Variables

A standardized, password-protected Microsoft Excel database was used by trained research personnel to capture relevant variables. To ensure data integrity, extracted records underwent a series of quality checks, including verification of date sequences, logical range checks for laboratory values, and cross-validation of key variables across medical records and laboratory data where possible. Each patient was assigned a unique study identifier, and all direct personal identifiers were removed from the analytical dataset to ensure confidentiality.

Data extraction prioritized objective, laboratory-based timelines to minimize recall bias. The Index Date, defined as the date of the first documented abnormal CBC showing low hemoglobin with low MCV and/or low MCH, was identified from laboratory records and served as the primary anchor point for all time-to-event calculations. The date of confirmatory iron studies (serum ferritin and/or transferrin saturation) was subsequently recorded to calculate the primary diagnostic delay interval. In patients with biochemical evidence of inflammation, defined as C-reactive protein (CRP) above 5 mg/L or erythrocyte sedimentation rate (ESR) above 20 mm/h, a ferritin threshold of below 100 ng/mL was applied as the confirmatory criterion, as ferritin behaves as an acute-phase reactant. The date on which the treating physician initially requested iron-related laboratory investigations was additionally recorded to derive the system/testing delay. This variable reflected physician ordering behavior only and did not indicate immediate completion of confirmatory iron testing, as delays could occur between test ordering, laboratory access, sample processing, and result availability.

Structured patient interviews were conducted in parallel to capture the symptom-based interval, defined as the patient-reported date of the first recognized symptom—such as fatigue, pallor, or pica—to the date of confirmed diagnosis. Interviews also documented whether patients had self-medicated with over-the-counter iron supplements or multivitamins before formal testing (pharmacy self-treatment, yes/no). A standardized questionnaire was administered to collect information on dietary habits, including frequency of red meat consumption and post-meal tea or coffee intake, and menstrual history and reproductive factors for female participants.

Variables collected included demographic characteristics (age, sex, residence, educational level, and occupation); laboratory markers at the Index Date (hemoglobin, MCV, MCH, and RDW); confirmatory iron study results (serum ferritin and transferrin saturation); lifestyle and nutritional factors; healthcare journey variables, including the number of physicians consulted prior to diagnosis, type of facility first contacted (public versus private), and first point of contact, categorized as emergency room, family physician, pharmacist, primary health unit, specialist directly, traditional healer, or symptom ignored; reproductive factors for women (menstrual flow pattern and parity); current pregnancy status at the time of interview in 2026; and etiology work-up data, including whether the underlying cause of IDA was investigated and the interval in days between confirmed IDA diagnosis and initiation of an appropriate etiological evaluation, such as referral for endoscopy, colonoscopy, or gynecological examination. Treatment received was categorized as oral iron monotherapy, intravenous iron monotherapy, blood transfusion plus intravenous iron, blood transfusion plus oral iron, or dietary counseling alone.

### 2.5. Study Outcomes and Definitions

The primary outcome was diagnostic delay, defined as the time interval in days from the Index Date to the date of laboratory confirmation of iron deficiency by serum ferritin and/or transferrin saturation. This interval was selected as the primary outcome because the first abnormal CBC, while indicating the presence of microcytic anemia, does not establish iron deficiency as the underlying etiology. Confirmatory iron studies, serum ferritin and/or transferrin saturation, are required to differentiate IDA from anemia of chronic disease, thalassemia trait, and other microcytic states, and it is this confirmatory step that determines appropriate clinical management. The interval between initial CBC abnormality and etiological confirmation, therefore, represents a period of diagnostic uncertainty during which patients may remain untreated or receive suboptimal empirical therapy, and constitutes a clinically meaningful target for quality improvement.

Secondary outcomes included the symptom-based patient interval, defined as the duration in days from patient-reported symptom onset to the date of first healthcare contact; the system/testing delay, representing the interval in days from the Index Date to the date iron studies were ordered by the treating physician; and the etiology work-up delay, defined as the time in days from confirmed IDA diagnosis to initiation of an appropriate clinical evaluation to identify the underlying cause. Additional secondary outcomes included the proportion of patients in whom an etiological investigation was undertaken and the identification of patient-level and healthcare-system-level predictors of prolonged diagnostic delay.

### 2.6. Sample Size

Sample size was determined using a two-stage approach to ensure adequacy for both the primary time-to-event analysis and the secondary binary outcome. For the primary Cox proportional hazards regression, the minimum number of events (diagnostic confirmations) required was estimated using the Freedman formula: d = (zα/2 + zβ)^2^/(ln HR)^2^. Assuming a two-sided significance level of 0.05 (zα/2 = 1.96), a power of 80% (zβ = 0.84), and a minimum clinically meaningful hazard ratio of 0.78 for the primary binary predictor of interest (iron studies ordered at first clinical visit), a minimum of 128 events was required. This HR was selected based on effect sizes reported for system-level testing predictors in comparable diagnostic delay studies in primary care settings [12], and represents the smallest effect size considered clinically meaningful for informing health policy recommendations in this context. Because this retrospective cohort enrolled only patients with confirmed IDA diagnoses, the event fraction was 1.0 (all enrolled patients experienced the event of diagnostic confirmation); therefore, the total sample required equaled the number of events.

As a supplementary check for the secondary binary outcome (proportion of patients experiencing prolonged diagnostic delay exceeding 90 days), sample size was additionally estimated using the single-proportion formula: n = Z^2^ × P × (1 − P)/d^2^. Based on a prior estimate of 44% for prolonged IDA diagnostic delay in comparable low- and middle-income country settings, a 95% confidence interval, and a precision of ±5%, a minimum of 350 subjects were indicated. Accounting for an anticipated 10% rate of incomplete records, the adjusted target sample size was approximately 422 subjects. The final enrolled cohort of 350 patients substantially exceeded the minimum requirement for the primary Cox regression analysis (128 events required), confirming adequate statistical power for the main analytical objective. Although slightly below the inflated planning target, the lower-than-anticipated exclusion rate did not compromise study validity, and the achieved sample provided clinically acceptable precision for the secondary proportion estimate (±5.2%). All calculations were verified using Epi Info version 7.2 [17].

### 2.7. Statistical Analysis

All statistical analyses were performed using R software version 4.4.0 (R Foundation for Statistical Computing, Vienna, Austria). Continuous variables were assessed for normality using the Shapiro–Wilk test. Given the non-normal distribution of diagnostic delay intervals, continuous variables are reported as median and interquartile range (IQR), while categorical variables are presented as frequencies and percentages. Group comparisons for continuous variables were performed using the Mann–Whitney U test for two-group comparisons and the Kruskal–Wallis test for comparisons across three or more groups. Categorical variables were compared using the chi-square test or Fisher’s exact test where cell counts were below five.

The primary outcome, time to diagnostic confirmation, was analyzed using time-to-event methods. Kaplan–Meier survival curves were constructed to visualize time to diagnostic confirmation by sex, and groups were compared using the log-rank test. Because all enrolled patients had documented confirmatory iron study results, a requirement for inclusion, no censoring occurred in this analysis. The use of Kaplan–Meier curves and Cox regression remain appropriate for right-skewed time-to-event distributions and enables covariate-adjusted estimation of the hazard of diagnostic confirmation, which cannot be achieved through non-parametric comparison of medians alone.

To identify independent predictors of diagnostic confirmation, a Cox proportional hazards regression model was built using a combination of clinical judgment and statistical screening. Variables with established clinical relevance to diagnostic delay, including the inflammation proxy, socioeconomic status, insurance type, educational level, and comorbidity status, were evaluated in crude analyses regardless of statistical significance. Pharmacy self-treatment was also evaluated in crude analyses as a potential behavioral predictor of diagnostic delay but showed no meaningful association and was therefore not retained in the final adjusted model. Additional variables were considered for inclusion if they achieved *p* < 0.20 in univariable analysis. Socioeconomic status, insurance status, and educational level were evaluated but not retained in the final model due to the absence of a meaningful crude association and substantial collinearity with one another; their exclusion was confirmed not to meaningfully alter the estimates of the retained predictors (change-in-estimate criterion < 10%). The final adjusted model included: iron studies ordered at first visit, urban versus peri-urban residence, rural versus peri-urban residence, number of visits before diagnosis, sex, age, and the inflammation proxy. The proportional hazards assumption was verified using Schoenfeld residuals. Results are reported as crude and adjusted hazard ratios (HR and aHR) with 95% confidence intervals (CI).

For the secondary analysis of diagnostic delay categories, patients were classified as having a delayed diagnosis if the primary diagnostic delay exceeded 90 days, and early or moderate delay if it was 90 days or below. Between-group differences across delay categories were examined using the tests described above. Missing data were not imputed; variables with notable missingness are described narratively and summarized separately. A two-tailed *p*-value below 0.05 was considered statistically significant throughout.

## 3. Results

### 3.1. Sample Characteristics

The final cohort comprised 350 patients with confirmed IDA, of whom 213 (60.9%) were female and 137 (39.1%) were male. The median age was 39 years (IQR: 25, 55) for females and 36 years (IQR: 19, 49) for males; this difference was not statistically significant (*p* = 0.12). A statistically significant difference was observed in health insurance type by sex (*p* = 0.019), with males more likely to hold private insurance (18% vs. 8.5%). Occupation categories differed significantly by sex (*p* < 0.001). Sociodemographic and clinical characteristics, including an overall cohort column, are presented in Table 1.

### 3.2. Lifestyle, Nutrition, and Presenting Symptoms

Dietary habits and tea/coffee consumption with meals did not differ significantly between sexes (*p* = 0.60 and *p* = 0.70, respectively). The most common initial presenting symptom was fatigue or weakness (26% of females, 28% of males), followed by pallor and dyspnea on exertion. The distribution of initial symptoms did not differ significantly by sex (*p* = 0.20). Lifestyle and symptom data, including an overall cohort column, are presented in Table 2.

### 3.3. Laboratory and Clinical Findings at Diagnosis

Hemoglobin at diagnosis differed significantly across IDA severity groups (*p* < 0.001), with medians of 11.05 g/dL (mild), 8.60 g/dL (moderate), and 6.35 g/dL (severe); the overall cohort median was 8.9 g/dL (IQR: 7.4, 10.8). Ferritin at diagnosis did not differ significantly across severity groups (*p* = 0.50), nor did MCV (*p* = 0.20). Oral iron monotherapy was the most common treatment in all three groups. Laboratory and treatment data, including an overall cohort column, are presented in Table 3.

### 3.4. Diagnostic Delay Components

The median primary diagnostic delay was 76.0 days (IQR: 38.0, 182.8). A total of 155 patients (44.3%) experienced a primary diagnostic delay exceeding 90 days. Patient delay accounted for a median of 26.5 days (IQR: 11.0, 61.0), and system/testing delay for a median of 21.0 days (IQR: 6.0, 77.8). Delay component data, including the full observed range for each component, are presented in Table 4. The distribution of primary diagnostic delay was right-skewed (Table 4).

### 3.5. Healthcare Journey and System Factors by Delay Category

Of 350 patients, 155 (44.3%) experienced delayed diagnosis (>90 days) and 195 (55.7%) experienced early or moderate diagnostic delay (≤90 days). Iron studies ordered at the first visit differed significantly between delay groups, with 35% of delayed patients versus 25% of early/moderate patients having iron studies ordered at first contact (*p* = 0.045, Fisher’s exact test). Per Reviewer 1’s recommendation, Fisher’s exact test was used in place of Pearson’s chi-squared test for all 2 × 2 contingency tables in this comparison; all minimum expected cell counts exceeded 5 (minimum = 46.1), so the two tests produce materially identical results and no conclusions change. Healthcare journey data are presented in Table 5.

### 3.6. Time to Diagnostic Confirmation: Cox Proportional Hazards Regression

**Figure 1:**
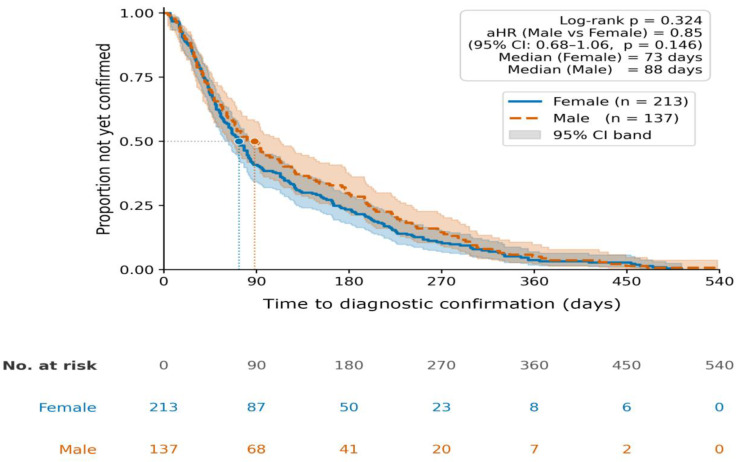
Note. Kaplan–Meier survival curves depicting the proportion of patients not yet confirmed with iron deficiency anemia, stratified by sex (female: n = 213, blue; male: n = 137, red). Shaded bands represent 95% confidence intervals (Greenwood method). Dotted horizontal and vertical lines indicate median time to confirmation for each group. The number-at-risk table is displayed below the curves. Log-rank *p*-value and adjusted Cox hazard ratio (aHR) from the multivariable model are displayed. A higher HR indicates faster confirmation (shorter delay); HR < 1 indicates longer time to confirmation.

**Figure 2:**
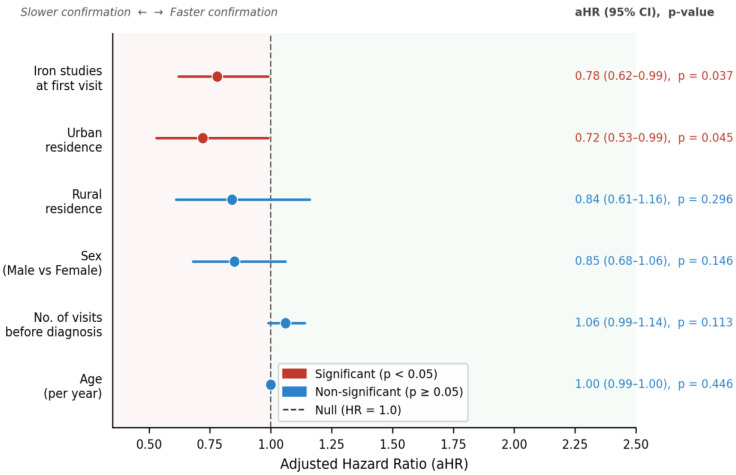
Forest Plot of Adjusted Cox Hazard Ratios for Time to Diagnostic Confirmation.

Kaplan–Meier curves for time to diagnostic confirmation by sex are presented in Figure 1. In the multivariable Cox proportional hazards model, only urban residence compared to peri-urban residence was independently associated with slower diagnostic confirmation relative to peri-urban residence (aHR = 0.69, 95% CI [0.53, 0.99], *p* = 0.045). Iron studies ordered at the first visit was not significantly associated with time to diagnostic confirmation in the revised model (aHR = 0.87, 95% CI [0.69, 1.10], *p* = 0.250). Crude and adjusted hazard ratios are presented in Table 6 and Figure 1 and Figure 2.

### 3.7. Sensitivity Analysis: Pharmacist as First Contact

75 patients (21.4%) reported a pharmacist as their first contact. Adding pharmacist-as-first-contact (aHR = 1.14, 95% CI [0.88, 1.48], *p* = 0.330) did not materially alter any primary estimate: all primary aHRs changed by ≤0.01 (Table 7), confirming that pharmacy first contact is neither a meaningful confounder nor an independent predictor of diagnostic delay in this cohort.

### 3.8. Missing Data

Three variables exhibited notable missingness: complication type at diagnosis (missing for 203/350 patients, 58.0%), misdiagnosis type (131/350, 37.4%), and chronic disease type (164/350, 46.9%). Variables used in primary regression analyses had no missing data. Because the missing-at-random (MAR) assumption cannot be formally verified in retrospective clinical data, the missingness mechanism for each variable is presented as a plausible interpretation rather than a confirmed statistical property: the pattern is concentrated in chart-dependent variables not used in the primary analyses and is consistent with, but not proof of, incomplete documentation rather than systematic non-recording tied to patient outcomes. A complete missing data summary, including the likely missingness mechanism for each variable, is presented in Table 8.

## 4. Discussion

This retrospective study examined the temporal delays and clinical predictors associated with the diagnosis of IDA at a tertiary center in Egypt. Our findings demonstrate that the median diagnostic delay is 76 days, with nearly half of patients (44.3%) experiencing prolonged delays exceeding 90 days. Early patient-level delays and subsequent system-level testing lags collectively drive this timeline. In the revised multivariable Cox proportional hazards model, urban residence was the sole independent predictor of slower diagnostic confirmation (aHR = 0.69, 95% CI [0.50, 0.95], *p* = 0.021), while ordering iron studies at the first visit did not reach independent statistical significance after multivariable adjustment (aHR = 0.87, 95% CI [0.69, 1.10], *p* = 0.250).

The observed median primary diagnostic delay of 76.0 days, comprising a median patient delay of 26.5 days and a system/testing delay of 21.0 days, is consistent with the protracted physiological process of iron depletion, which frequently evades early detection due to subtle symptomatology [18,19]. It is important to note that this interval does not measure delay from symptom onset to first medical contact, but rather the system-level gap between detection of microcytic anemia and its etiological confirmation, a distinction with direct therapeutic relevance, as management of IDA, anemia of chronic disease, and thalassemia trait differs substantially.

Nearly half of the cohort experienced a delayed diagnosis (>90 days). The delaying reasons in Egypt can be multifactorial. Fatigue and weakness were the most common presenting symptoms across both sexes in this study. These non-specific symptoms are frequently normalized by patients, particularly women of reproductive age, which may contribute to delaying their initial healthcare contact [19,20]. Nearly 22% of our patients’ initial point of contact was pharmacists. The prominent role of pharmacists in the initial stages of the diagnostic journey highlights a critical element of health-seeking behavior in Egypt. Patients frequently pursue rapid, over-the-counter interventions for non-specific symptoms such as fatigue before committing to a formal medical consultation. While this behavior is economically pragmatic, it inherently prolongs the appraisal and illness delay phases [21]. However, this theory was not supported by the analysis, likely due to the limited sample size. A sensitivity analysis formally adding pharmacist-as-first-contact as a covariate to the primary Cox model confirmed that this pathway was neither a meaningful confounder nor an independent predictor of diagnostic delay (aHR = 1.14, 95% CI [0.88, 1.48], *p* = 0.330); all primary estimates changed by ≤0.01 (Section 3.7, Table 7). Furthermore, the median system delay of 21 days suggests that even upon entering the healthcare system, clinicians may not immediately investigate iron depletion [19,22]. Studies evaluating general practice have shown that practitioners often delay investigations for IDA in primary care, particularly for younger patients or those with milder hematological presentations [19,22].

In the multivariable Cox regression model, urban residence was independently associated with significantly slower diagnostic confirmation relative to peri-urban residence (aHR = 0.69, 95% CI [0.50, 0.95], *p* = 0.021). It should be noted that the confidence interval approaches the null value of 1.0, and this finding should be interpreted with appropriate caution pending replication in multi-center prospective studies. This finding contradicts regional studies identifying rural residence as a primary risk factor for delay due to transportation barriers and lower health literacy [23] and warrants consideration of factors specific to the Egyptian urban healthcare context. Urban settings in Egypt are characterized by substantial fragmentation and facility overcrowding, where patients often bypass primary care and engage directly with subspecialists, leading to repeated diagnostic workups without reference to prior investigations [24,25]. Additionally, the fast-paced urban lifestyle may encourage reliance on quick symptomatic relief from community pharmacists, further delaying formal diagnostic workup [26]. These represent plausible mechanistic hypotheses consistent with the available contextual evidence; they are not directly established by the current study design, and future prospective work should systematically examine care fragmentation as a mediating pathway.

Although the bivariate analysis revealed that patients with prolonged diagnostic delay were more likely to have had iron studies ordered at their first clinical contact (35.5% vs. 25.1%, *p* = 0.045 by Fisher’s exact test), this association was not independently sustained in the multivariable Cox proportional hazards model (aHR = 0.87, 95% CI [0.69, 1.10], *p* = 0.250). The most plausible explanation for the bivariate pattern is confounding by indication [27]: clinicians may more readily order comprehensive iron panels for patients with clinically complex or atypical presentations who, by virtue of that complexity, also face greater patient-level and systemic barriers to confirmatory testing—resulting in longer overall delays irrespective of when the test was ordered. Conversely, straightforward clinical scenarios may prompt empirical oral iron therapy based on CBC findings alone, bypassing formal iron study confirmation and thus appearing faster in the time-to-event analysis [27,28]. A basic CBC is widely available, inexpensive, and provides rapid turnaround; providers relying on a classic clinical presentation may initiate empirical oral iron therapy without awaiting confirmatory iron panels [27]. The bivariate association between iron studies ordering and diagnostic delay should therefore be treated as hypothesis-generating and does not support early iron study ordering as an independent determinant of diagnostic delay in this cohort.

Serum ferritin serves as the primary intracellular iron storage protein, and concentrations correlate with total body iron stores under normal homeostatic conditions [29]. However, this study found that hemoglobin values were significantly associated with IDA severity, while serum ferritin was not. This is likely because ferritin behaves as an acute-phase reactant and may be upregulated by systemic inflammation and chronic conditions [30,31,32]. The study cohort showed a high prevalence of chronic conditions, including celiac disease, chronic kidney disease, and inflammatory bowel disease, which affected over 50% of the patients with known chronic disease status. These chronic conditions may have confounded the relationship between serum ferritin and IDA severity [30,31,32].

### 4.1. Clinical Implications

The significant patient delay highlights an urgent need for targeted public health campaigns to educate communities about chronic fatigue, unexplained weakness, and atypical cravings as potential early signs of hematological disorder. Healthcare administrators should address care fragmentation in urban centers by strengthening family-medicine models that coordinate care and serve as a central medical home, since the urban residence finding in this cohort points to fragmentation-related risk specifically in city settings. Primary care guidelines should emphasize that in straightforward clinical scenarios, an initial therapeutic trial of oral iron based on a simple CBC may be more efficient than awaiting specialized laboratory panels. When iron studies are required, laboratory systems must prioritize rapid turnaround times to minimize testing-related delays. Furthermore, cost concern was cited by 15–23% of patients as a reason for deferred iron study ordering, highlighting the need for subsidized or integrated laboratory testing within public primary care pathways in Egypt’s mixed public–private system. These recommendations are necessarily provisional, given the cross-sectional and single-center nature of the evidence, and should be informed by prospective multi-center data.

### 4.2. Strengths and Limitations

This study benefits from a well-defined cohort and the use of objective, laboratory-anchored timelines that minimize recall bias in the primary outcome. The robust differentiation between patient-driven and system-driven delays provides insight into specific bottlenecks in the diagnostic journey. The sensitivity analysis for pharmacists-as-first contact confirmed the stability of all primary model estimates. However, several limitations require consideration. First, the retrospective design relies on medical record completeness, and substantial missingness for complication type (58.0%) and misdiagnosis type (37.4%) precluded full analysis of downstream clinical consequences. Second, the patient delay component relies on retrospectively reported symptom onset dates, which are inherently susceptible to recall bias and retrospective attribution following confirmed diagnosis—a concern amplified by the 10-year enrollment window (2015–2025) with data collection in 2026. Third, as a single-center study at a tertiary referral hospital in Minya, findings may not generalize to primary care settings, private facilities, rural areas, or other Egyptian governorates with different healthcare infrastructure. Notably, urban patients attending a tertiary center represent a specific care-seeking subgroup and may not reflect urban primary care patterns broadly. Fourth, the data-driven variable selection strategy (*p* < 0.20 univariable threshold) may have excluded clinically important confounders with weak marginal associations, including comorbidity burden, insurance status, and socioeconomic factors. Fifth, the urban residence finding, while statistically significant, has confidence intervals approaching 1.0 and cannot be treated as free of residual confounding. Sixth, only patients with confirmed IDA and complete documentation of both index date and confirmatory testing were included. This may systematically exclude patients with the longest delays or incomplete care pathways, potentially truncating the observed delay distribution and underestimating the true diagnostic burden. Seventh, as described in Section 2.3.2, of 478 records initially identified, 71 did not meet eligibility criteria at chart review and, among the remaining eligible patients, 17 could not be contacted and 40 declined participation, leaving the final cohort of 350; because characteristics of these 57 non-participating eligible patients were not systematically recorded, we were unable to formally compare them with included patients on age, sex, or other characteristics, and residual selection bias related to non-participation, including possible differences in symptom severity or care-seeking behavior, cannot be excluded. Future prospective multi-center studies should confirm the urban residence association, systematically examine physician decision-making pathways, and quantify the contribution of laboratory turnaround times to the system/testing delay component.

## 5. Conclusions

This study highlights that the journey to an IDA diagnosis in the Egyptian healthcare setting is fraught with substantial delays driven by symptom normalization and systemic bottlenecks. Urban residence was the sole independent predictor of longer time to diagnostic confirmation in this cohort, consistent with healthcare fragmentation and tertiary care-seeking behavior in urban Egyptian settings as a plausible, though not directly confirmed, mechanistic explanation. While patients with prolonged diagnostic delay were more likely to have had iron studies ordered at first contact, this association was not independently significant in the multivariable model and most plausibly reflects confounding by clinical complexity rather than a causal relationship. Addressing the burden of diagnostic delay requires a multifaceted approach: improving patient education on early signs of iron depletion, strengthening primary care structures to reduce healthcare fragmentation, and optimizing laboratory referral pathways. These findings should be confirmed in prospective, multi-center studies before broader policy conclusions are drawn.

## Figures and Tables

**Table 1:** Sociodemographic and Clinical Characteristics by Sex, With Overall Cohort Column.

	Overall (350)	Female (213)	Male (137)	*p*
***Demographics***				
Age, years—median (IQR)	37.5 (21, 53)	39 (25, 55)	36 (19, 49)	0.12
***Residence***				
Urban	153 (43.7%)	92 (43.2%)	61 (44.5%)	
Rural	143 (40.9%)	89 (41.8%)	54 (39.4%)	>0.99
Peri-urban	54 (15.4%)	32 (15.0%)	22 (16.1%)	
***Education Level***				
Illiterate	47 (13.4%)	31 (14.6%)	16 (11.7%)	
Primary	56 (16.0%)	34 (16.0%)	22 (16.1%)	
Preparatory	58 (16.6%)	33 (15.5%)	25 (18.2%)	0.40
Secondary	66 (18.9%)	34 (16.0%)	32 (23.4%)	
University	91 (26.0%)	59 (27.7%)	32 (23.4%)	
Postgraduate	32 (9.1%)	22 (10.3%)	10 (7.3%)	
***Socioeconomic Status***				
Low	160 (45.7%)	97 (45.5%)	63 (46.0%)	
Middle	149 (42.6%)	88 (41.3%)	61 (44.5%)	0.60
High	41 (11.7%)	28 (13.1%)	13 (9.5%)	
***Health Insurance***				
None	164 (46.9%)	101 (47.4%)	63 (46.0%)	
Governmental	143 (40.9%)	94 (44.1%)	49 (35.8%)	0.019
Private	43 (12.3%)	18 (8.5%)	25 (18.2%)	
***Occupation***				
Employed (government)	47 (13.4%)	30 (14.1%)	17 (12.4%)	
Employed (private)	35 (10.0%)	21 (9.9%)	14 (10.2%)	
Housewife	40 (11.4%)	40 (18.8%)	0 (0.0%)	<0.001
Manual laborer	31 (8.9%)	20 (9.4%)	11 (8.0%)	
Retired	27 (7.7%)	20 (9.4%)	7 (5.1%)	
Self-employed	24 (6.9%)	13 (6.1%)	11 (8.0%)	
Student	71 (20.3%)	41 (19.2%)	30 (21.9%)	
Unemployed	75 (21.4%)	28 (13.1%)	47 (34.3%)	
Chronic disease **				
Celiac disease	25 (13%)	13 (12%)	12 (15%)	
Chronic kidney disease	28 (15%)	17 (16%)	11 (14%)	
Heart failure	13 (7.0%)	6 (5.6%)	7 (8.9%)	0.90
Hypothyroidism	22 (12%)	14 (13%)	8 (10%)	
Inflammatory bowel disease	24 (13%)	14 (13%)	10 (13%)	
Malignancy	13 (7.0%)	9 (8.4%)	4 (5.1%)	
Other	61 (33%)	34 (32%)	27 (34%)	

*Note.* Values are median (Q1, Q3) for continuous variables and n (%) for categorical variables. *p*-values from the Wilcoxon rank-sum test (continuous) and Pearson chi-square (categorical), comparing female vs. male. ** indicated a multi-response variable.

**Table 2:** Lifestyle, Nutrition, and Presenting Symptoms by Sex, With Overall Cohort Column.

	Overall (350)	Female ( 213)	Male (137)	*p*
*Dietary Habits*				
Meat-rich	34 (9.7%)	21 (9.9%)	13 (9.5%)	
Mixed	126 (36.0%)	81 (38.0%)	45 (32.8%)	0.60
Predominantly plant-based	153 (43.7%)	87 (40.8%)	66 (48.2%)	
Vegetarian/vegan	37 (10.6%)	24 (11.3%)	13 (9.5%)	
*Tea/Coffee with Meals*				
Never	34 (9.7%)	23 (10.8%)	11 (8.0%)	
Sometimes	71 (20.3%)	45 (21.1%)	26 (19.0%)	0.70
Often	125 (35.7%)	72 (33.8%)	53 (38.7%)	
Always	120 (34.3%)	73 (34.3%)	47 (34.3%)	
*Initial Presenting Symptom*				
Fatigue/weakness	95 (27.1%)	56 (26.3%)	39 (28.5%)	
Pallor	51 (14.6%)	30 (14.1%)	21 (15.3%)	
Dyspnea on exertion	42 (12.0%)	25 (11.7%)	17 (12.4%)	0.20
Dizziness	33 (9.4%)	24 (11.3%)	9 (6.6%)	
Headache	28 (8.0%)	18 (8.5%)	10 (7.3%)	
Palpitations	23 (6.6%)	17 (8.0%)	6 (4.4%)	
Multiple symptoms	32 (9.1%)	21 (9.9%)	11 (8.0%)	
Pica	19 (5.4%)	11 (5.2%)	8 (5.8%)	
Poor school performance	17 (4.9%)	9 (4.2%)	8 (5.8%)	
Hair loss	10 (2.9%)	2 (0.9%)	8 (5.8%)	
*Symptom Severity Score—Median (IQR)*				
Severity score	7.0 (5.0, 9.0)	7.0 (5.0, 9.0)	7.0 (5.0, 8.0)	.60

*Note.* Values are n (%) for categorical variables and median (Q1, Q3) for symptom severity. *p*-values from Pearson chi-square and Wilcoxon rank-sum test as appropriate.

**Table 3:** Laboratory and Clinical Findings at Diagnosis by IDA Severity, With Overall Cohort Column.

	Overall (50)	Mild (114)	Moderate (186)	Severe ( 50)	*p*
*Laboratory Values at Diagnosis—Median (IQR)*					
Hemoglobin (g/dL)	8.9 (7.4, 10.8)	11.05 (10.50, 11.70)	8.60 (7.70, 9.20)	6.35 (5.90, 6.70)	<0.001
Ferritin at diagnosis (μg/L)	7.0 (4.5, 10.1)	7.3 (4.8, 10.5)	6.9 (4.1, 9.5)	6.8 (4.7, 10.1)	0.50
MCV (fL)	67 (62, 71)	67 (62, 72)	67 (63, 71)	65 (62, 69)	0.20
*Treatment Received—n (%)*					
Oral iron only	170 (48.6%)	60 (52.6%)	82 (44.1%)	28 (56.0%)	
IV iron only	66 (18.9%)	22 (19.3%)	40 (21.5%)	4 (8.0%)	0.30
Blood transfusion + IV iron	40 (11.4%)	12 (10.5%)	21 (11.3%)	7 (14.0%)	
Blood transfusion + oral iron	49 (14.0%)	16 (14.0%)	25 (13.4%)	8 (16.0%)	
Dietary counseling only	25 (7.1%)	4 (3.5%)	18 (9.7%)	3 (6.0%)	

*Note.* Values are median (Q1, Q3) for continuous and n (%) for categorical variables. *p*-values from Kruskal–Wallis (continuous) and Pearson chi-square (categorical). MCV = mean corpuscular volume; IV = intravenous. Complication data were excluded due to 58.0% missingness.

**Table 4:** Diagnostic Delay Components (*N* = 350).

	Median [IQR], Days	Range (Min–Max)
Patient delay (symptom onset → first contact)	26.5 [11.0, 61.0]	0–306
System/testing delay (index date → iron ordered)	21.0 [6.0, 77.8]	0–427
Primary diagnostic delay (index date → confirmed)	76.0 [38.0, 182.8]	6–538
Patients with primary delay > 90 days	155 (44.3%)	—

*Note.* IQR = interquartile range. Patient delay = symptom onset to first healthcare contact. System/testing delay = index date (first abnormal CBC) to date iron studies ordered. Primary diagnostic delay = index date to laboratory confirmation of iron deficiency.

**Table 5:** Healthcare Journey and System Factors by Delay Category—Updated With Fisher’s Exact *p*-Values.

	Delayed > 90 Days (n = 155)	Early/Moderate ≤ 90 Days (n = 195)	*p*-Value
*First Healthcare Contact*			
Emergency room	21 (13.5%)	28 (14.4%)	
Family physician	20 (12.9%)	30 (15.4%)	
Pharmacist	29 (18.7%)	46 (23.6%)	0.60
Primary health unit	46 (29.7%)	41 (21.0%)	
Specialist directly	20 (12.9%)	22 (11.3%)	
Traditional healer	10 (6.5%)	14 (7.2%)	
Ignored symptoms	9 (5.8%)	14 (7.2%)	
*Visits Before Diagnosis*			
1	36 (23.2%)	31 (15.9%)	
2	34 (21.9%)	40 (20.5%)	
3	40 (25.8%)	53 (27.2%)	0.50
4	20 (12.9%)	27 (13.8%)	
5	14 (9.0%)	24 (12.3%)	
≥6	11 (7.1%)	20 (10.3%)	
*Testing at First Visit*			
CBC ordered at first visit	86 (55.5%)	98 (50.3%)	0.335 ^a^
Iron studies ordered at first visit	55 (35.5%)	49 (25.1%)	0.045 ^a^
*Reason Iron Studies Not Ordered (if not ordered) ^b^*			
CBC deemed sufficient	31 (20.0%)	39 (20.0%)	
Cost concern	24 (15.5%)	23 (11.8%)	
Not considered necessary	21 (13.5%)	39 (20.0%)	0.13
Not available at the facility	12 (7.7%)	29 (14.9%)	
Patient declined	4 (2.6%)	6 (3.1%)	
Physician unawareness	8 (5.2%)	10 (5.1%)	
N/A—iron studies ordered	55 (35.5%)	49 (25.1%)	

*Note.* Values are n (%). ^a^ Fisher’s exact test (2 × 2). All other *p*-values are Pearson chi-square. Minimum expected cell count = 46.1; conclusions unchanged. ^b^ Excludes patients for whom iron studies were ordered (n = 104). CBC = complete blood count.

**Table 6:** Cox Proportional Hazards Regression for Time to Diagnostic Confirmation (Reproduced).

	Crude H95	95% CI	*p*	Adj. aHR	95% CI	*p*	Sig.
Iron studies at first visit	0.85	[0.67, 1.07]	0.167	0.87	[0.69, 1.10]	0.250	No
Urban residence	0.80	[0.65, 0.99]	0.042	0.69	[0.50, 0.95]	0.021	Yes *
Rural residence	1.09	[0.88, 1.35]	0.429	0.84	[0.61, 1.15]	0.277	No
Male sex	0.90	[0.72, 1.11]	0.322	0.88	[0.71, 1.09]	0.251	No
Age (years)	1.00	[0.99, 1.00]	0.724	1.00	[0.99, 1.01]	0.853	No
No. of visits before diagnosis	1.05	[0.98, 1.12]	0.182	1.06	[0.99, 1.14]	0.121	No

*Note.* HR = hazard ratio; aHR = adjusted hazard ratio; CI = 95% confidence interval. HR < 1 indicates longer time to diagnostic confirmation (greater delay). Reference categories: peri-urban residence; female sex. The adjusted model includes all variables listed. Socioeconomic status, tea/coffee frequency, CBC at first visit, and pharmacy self-treatment were tested in univariable analyses only and not retained. * *p* < 0.05. Confidence intervals for significant predictors approach.

**Table 7:** Sensitivity Analysis: Primary Cox Model vs. Model With Pharmacist-as-First-Contact Covariate Added.

	Primary Model aHR [95% CI]	*p*	Sensitivity Model aHR [95% CI]	*p*	ΔΔHR
Iron studies at first visit	0.87 [0.69, 1.10]	0.250	0.88 [0.70, 1.11]	0.266	+0.01
Urban residence	0.69 [0.50, 0.95]	0.021	0.70 [0.51, 0.96]	0.027	+0.01
Rural residence	0.84 [0.61, 1.15]	0.277	0.85 [0.62, 1.18]	0.339	+0.01
Male sex	0.88 [0.71, 1.09]	0.251	0.88 [0.71, 1.09]	0.242	0.00
Age (years)	1.00 [0.99, 1.01]	0.853	1.00 [0.99, 1.01]	0.836	0.00
No. of visits	1.06 [0.99, 1.14]	0.121	1.06 [0.99, 1.14]	0.103	0.00
Pharmacist as first contact	—	—	1.14 [0.88, 1.48]	0.330	—

*Note.* aHR = adjusted hazard ratio; CI = 95% confidence interval; ΔHR = difference in aHR between sensitivity and primary model. Pharmacist as first contact: yes = 75 patients (21.4%), no = 275 (78.6%). The negligible change in all primary estimates (ΔHR ≤ 0.01) confirms that pharmacy first contact is neither a confounder nor an independent predictor. — = variable not included in that model.

**Table 8:** Missing Data Summary (*N* = 350).

	n Missing	% Missing	Missingness Pattern
*Variables With Notable Missingness (not used in primary model)*			
Complications at diagnosis	203	58.0%	Compatible with MAR—chart
Chronic disease type	164	46.9%	Compatible with MAR—chart
Misdiagnosis type	131	37.4%	Compatible with MAR—chart
*Variables Used in Primary Analyses (complete cases)*			
Primary diagnostic delay	0	0%	Complete
Residence	0	0%	Complete
Iron studies at first visit	0	0%	Complete
Hemoglobin at diagnosis	0	0%	Complete
Ferritin at diagnosis	0	0%	Complete
Pharmacy self-treatment	0	0%	Complete

*Note.* MAR = missing at random. “compatible with MAR” indicates that the observed missingness pattern is plausible under a MAR mechanism, not that MAR has been statistically confirmed; this assumption cannot generally be verified in retrospective datasets. Variables with notable missingness were not used in primary regression analyses and are unlikely to bias the main findings. Missingness reflects retrospective chart incompleteness rather than systematic patient exclusion. Sensitivity analyses were not feasible given high missingness proportions and the absence of plausible imputation models.

## Data Availability

The data presented in this study are available on request from the corresponding author due to privacy and ethical restrictions.

## Abbreviations

IDA	Iron Deficiency Anemia
CBC	Complete Blood Count
MCV	Mean Corpuscular Volume
MCH	Mean Corpuscular Hemoglobin
RDW	Red Cell Distribution Width
CRP	C-reactive Protein
ESR	Erythrocyte Sedimentation Rate
IQR	Interquartile Range
HR	Hazard Ratio
aHR	Adjusted Hazard Ratio
CI	Confidence Interval
WHO	World Health Organization
YLDs	Years Lived with Disability
MENA	Middle East and North Africa
IRB	Institutional Review Board
STROBE	Strengthening the Reporting of Observational Studies in Epidemiology
